# Ontogenetic Oxycodone Exposure Affects Early Life Communicative Behaviors, Sensorimotor Reflexes, and Weight Trajectory in Mice

**DOI:** 10.3389/fnbeh.2021.615798

**Published:** 2021-02-22

**Authors:** Elena Minakova, Simona Sarafinovska, Marwa O. Mikati, Kia M. Barclay, Katherine B. McCullough, Joseph D. Dougherty, Ream Al-Hasani, Susan E. Maloney

**Affiliations:** ^1^Department of Pediatrics, Washington University in St. Louis, St. Louis, MO, United States; ^2^Department of Genetics, Washington University in St. Louis, St. Louis, MO, United States; ^3^Department of Psychiatry, Washington University in St. Louis, St. Louis, MO, United States; ^4^Medical Scientist Training Program, Washington University in St. Louis, St. Louis, MO, United States; ^5^Division of Biology and Biomedical Sciences, Washington University in St. Louis, St. Louis, MO, United States; ^6^Department of Anesthesiology, Washington University in St. Louis, St. Louis, MO, United States; ^7^Washington University Pain Center, Washington University in St. Louis, St. Louis, MO, United States; ^8^Center for Clinical Pharmacology, St. Louis College of Pharmacy, University of Health Sciences and Pharmacy, St. Louis, MO, United States; ^9^Intellectual and Developmental Disabilities Research Center, Washington University In St. Louis, St. Louis, MO, United States

**Keywords:** opioid, behavior, *in utero*, post-natal, oxycodone, neonatal abstinence syndrome

## Abstract

Nationwide, opioid misuse among pregnant women has risen four-fold from 1999 to 2014, with commensurate increase in neonates hospitalized for neonatal abstinence syndrome (NAS). NAS occurs when a fetus exposed to opioids *in utero* goes into rapid withdrawal after birth. NAS treatment via continued post-natal opioid exposure has been suggested to worsen neurodevelopmental outcomes. We developed a novel model to characterize the impact of *in utero* and prolonged post-natal oxycodone (Oxy) exposure on early behavior and development. Via subcutaneous pump implanted before breeding, C57BL/6J dams were infused with Oxy at 10 mg/kg/day from conception through pup-weaning. At birth, *in utero* oxy-exposed pups were either cross-fostered (paired with non-Oxy exposed dams) to model opioid abstinence (*in utero* Oxy) or reared by their biological dams still receiving Oxy to model continued post-natal opioid exposure (prolonged Oxy). Offspring from vehicle-exposed dams served as cross-fostered (*in utero* Veh) or biologically reared (prolonged Veh) controls. *In utero* Oxy exposure resulted in sex-dependent weight reductions and altered spectrotemporal features of isolation-induced ultrasonic vocalization (USV). Meanwhile, prolonged Oxy pups exhibited reduced weight and sex-differential delays in righting reflex. Specifically, prolonged Oxy female offspring exhibited increased latency to righting. Prolonged Oxy pups also showed decreases in number of USV calls and changes to spectrotemporal USV features. Overall, ontogenetic Oxy exposure was associated with impaired attainment of gross and sensorimotor milestones, as well as alterations in communication and affective behaviors, indicating a need for therapeutic interventions. The model developed here will enable studies of withdrawal physiology and opioid-mediated mechanisms underlying these neurodevelopmental deficits.

## Introduction

In the past two decades, illicit drug use and prescription opioid use in the United States have risen to epidemic proportions, with the United States Department of Health declaring a public health emergency in 2017. The United States Department of Health states that 46,802 people died from opioid overdose in 2018 and an estimated 2 million people have an opioid use disorder (ASPA, 2017). The public health crisis is largely driven by increased misuse of the prescription opioids hydrocodone, oxycodone (Oxy), and methadone (Kenan et al., 2012; Haight, 2018).

As a result of the opioid epidemic, the national prevalence of opioid use disorder among pregnant women has more than quadrupled, from 1.5 to 6.5 per 1,000 deliveries, from 1999 through 2014 (Haight, 2018). Consequently, there has been a significant increase in the number of neonates hospitalized for neonatal abstinence syndrome (NAS), a constellation of withdrawal symptoms affecting the nervous system, gastrointestinal tract, and respiratory system following *in utero* exposure to opioids (Wiles et al., 2014). Currently, medical management of NAS involves keeping the infant swaddled in a low-stimulation environment with promotion of maternal-infant bonding (Wiles et al., 2014). In cases of moderate-severe NAS, neonatal withdrawal is managed by opioid replacement therapy to alleviate withdrawal symptomatology (Wiles et al., 2014). Overall, clinical studies have not addressed whether long-term neurobehavioral outcomes are improved by managing withdrawal or whether continued post-natal exposure to opioids and adjunct agents used for withdrawal management worsen long-term outcomes (Hudak et al., 2012).

Epidemiological evidence suggests that *in utero* opioid exposure is associated with lower birth weight and adverse neurodevelopmental outcomes in childhood, including cognitive deficits, attention deficit hyperactivity disorder (ADHD), aggression, impaired language development, and decreased social maturity (Hunt et al., 2008; Azuine et al., 2019; Conradt et al., 2019). However, large epidemiological studies evaluating long-term behavioral outcomes of children exposed to *in utero* opioids have been difficult to perform due to confounding environmental variables including genetic and epigenetic factors, quality of caregiving, continued parental substance abuse with its impact on the maternal-infant dyad, and other socioeconomic variables which can significantly affect neurodevelopmental outcomes (Lutz and Kieffer, 2013). Consequently, the development of ontogenetic rodent models of opioid exposure is necessary to enable investigation of the biological mechanisms mediating deficits as well as testing alternative treatment avenues for post-natal withdrawal.

To date, there have been a limited number of rodent studies evaluating early life developmental milestones following *in utero* opioid exposure. Current literature on early developmental effects of *in utero* opioid exposure in pre-clinical models demonstrates decreased birth weight following methadone and buprenorphine exposure (Kunko et al., 1996; Hung et al., 2013; Chiang et al., 2015). Increased latency to right has been observed following *in utero* morphine exposure and is suggestive of different classes of opioids having variable effects on developmental outcomes (Slamberová et al., 2005; Niu et al., 2009). Opioids exert their pharmacologic effects by activating the endogenous opioid system. While opioids are prescribed for their analgesic effects, acute activation of the μ-opioid receptor (MOR) by these medications has also been associated with feelings of euphoria, award reinforcement, and increased socio-emotional processing, which are linked to the drugs’ potential for misuse (Vanderschuren et al., 1995). Despite the rising incidence of Oxy misuse, there is a paucity of literature evaluating the effects of Oxy, a μ- and κ-agonist, on early developmental behaviors. κ-agonists are of particular interest because over-activation of κ-opioid receptors (KOR) by dynorphin upregulation has been implicated in withdrawal physiology and depressed mood in humans, along with decreased social play in juvenile rodents (Vanderschuren et al., 1995; Li et al., 2016). In addition, most rodent opioid exposure models begin exposure mid-pregnancy, which may explain inconsistently documented or absent developmental changes (Richardson et al., 2006). To address the above concerns, we are adopting an ontogenetic model in which opioid exposure spans preconception through early offspring development. This new model also enables us to better understand the ontogenetic impact of short- and long-term opioid exposure on early development in the absence of confounding factors present in clinical observational studies. Specifically, we evaluated the effects of *in utero* Oxy exposure on early developmental and behavioral outcomes in male and female offspring of C57BL/6J mouse dams. We implemented a cross-fostering approach that allows us to compare the neurodevelopmental impact of continued post-natal opioid exposure (prolonged Oxy) to the impact of exposure only until birth (*in utero* Oxy) by pairing opioid exposed pups with non-oxy exposed dams.

Overall, we observed differences in the spectrotemporal features of affective vocalizations and sex-based differences in weight gain trajectories in offspring exposed to *in utero* Oxy. Continued post-natal Oxy exposure (prolonged Oxy) further impacted weight, communicative behavior, and sensorimotor reflexes. Our findings suggest that pups with continued post-natal opioid exposure showed worse overall developmental outcomes compared to pups following opioid cessation at birth, which may have implications regarding the safety of continued opioid treatment as mitigation for clinical NAS symptomology.

## Materials and Methods

### Animals

#### Animal Ethics, Selection, and Welfare

All procedures using mice were approved by the Washington University Institutional Care and Use Committee and conducted in accordance with the approved Animal Studies Protocol. C57BL/6J mice (Jackson Laboratory, stock #: 000664) were housed in individually ventilated translucent plastic cages (IVC) measuring 36.2 × 17.1 × 13 cm (Allentown) with corncob bedding and *ad libitum* access to standard lab diet and water. Animals were kept at 12/12 h light/dark cycle, and room temperature (20–23°C) and relative humidity (50%) were controlled automatically.

**FIGURE 1:**
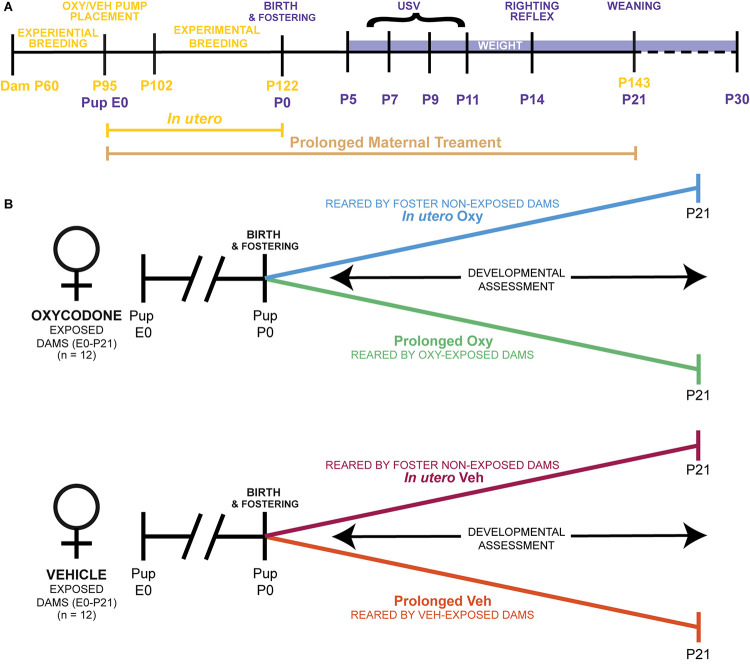
Ontogenetic model of prolonged and limited maternal Oxy exposure. **(A)** Schematic of the paradigm for maternal Oxy exposure, including duration of *in utero* and prolonged maternal treatment, dam ages at experimental manipulations in yellow, and pup age for behavioral tests in purple. **(B)** Outline of the four different experimental groups, including Oxy or Veh exposure, and rearing dam exposure status.

Adult male and female mice were used for breeding cohorts as described below. Sample sizes were determined by power analyses (*f* = 0.40, α = 0.05, 1-β = 0.80). A total of 24 dams were housed in pairs and randomly selected to receive either the Oxy or Vehicle (Veh) treatment infusion. In addition, another set of pair-housed, drug-naïve dams served as foster dams. The total sample size was 111 pups (Table 1). Since an inexperienced dam can exhibit poor maternal behavior with her first litter, all females were first bred to an age-matched male at post-natal day (P) 60. Following weaning of the first litter, treatment dams underwent surgical subcutaneous pump placement at P95 followed by a 1-week recovery period (Figure 1A). Afterward, each female dam was placed into an individual cage containing a male for breeding. Foster dams were bred at the same time and remained untreated throughout pregnancy. Following 20 days of co-habitation, cages were checked daily for pups. Upon detection, dam and litter were moved to a new cage, without the male, and culled to 6–8 pups per litter with equal males and females when possible. To evaluate the behavioral impact of early opioid cessation in the developing offspring, half of the litters (*in utero* Oxy and *in utero* Veh) were cross-fostered at this time to drug-naïve dams by removing the pups from their biological dam and transferring them to the nest of a lactating foster dam with two of her own pups of the same approximate age (Figure 1B; Lohmiller and Swing, 2006). The remaining litters were reared by the biological dam and exposed to post-natal vehicle (prolonged Veh) or Oxy (prolonged Oxy) through lactation (Figures 1A,B). To control for litter effects, each group included multiple, independent litters (Table 1). All mice were weaned at P21 and group-housed by sex with random assignment for drug/dam. A subset of the *in utero* Oxy mice required saline injections at P23-P25 due to skin tenting, hunched posture, and significant weight loss concerning for dehydration. Following saline injections, recovery was noted in two of the three affected mice with one associated mortality. Experimenters were all female and blinded to group designations during testing.

**TABLE 1:** Litter and group size, including number of pups at the level of litter, group, and experiment.

**Litter ID**	**Group**	**No. of pups**
1	Prolonged Oxy	5
2	Prolonged Oxy	4
3	Prolonged Oxy	3
4	Prolonged Oxy	6
5	Prolonged Oxy	6
Total:		24
6	*In utero* Oxy	5
7	*In utero* Oxy	6
8	*In utero* Oxy	6
9	*In utero* Oxy	6
10	*In utero* Oxy	6
Total:		29
11	Prolonged Veh	6
12	Prolonged Veh	6
13	Prolonged Veh	6
14	Prolonged Veh	6
15	Prolonged Veh	6
Total:		30
16	*In utero* Veh	5
17	*In utero* Veh	6
18	*In utero* Veh	3
19	*In utero* Veh	6
20	*In utero* Veh	5
21	*In utero* Veh	3
Total:		28
	Total pups:	111

#### Drug Dosage

The dosage of Oxy (Sigma-Aldrich, Saint Louis, MO, United States; Lot#: SLBX4974) administration was guided by previous literature with concentrations ranging from 0.5 to 33 mg/kg/day (Enga et al., 2016; Sithisarn et al., 2017; Zanni et al., 2020). Based on this dosage range, we generated our own dosage curve through continuous Oxy administration to pregnant dams at 5, 10, or 15 mg/kg/day using the subcutaneous Alzet 2006 model pump (Durect Corporation, Cupertino, CA, United States; Lot #: 10376-17). We chose the dose of 10 mg/kg/day administered at 0.15 ul/h to pregnant dams as increased concentrations at 15 mg/kg/day resulted in lower litter success rate (vehicle, 5 and 10 mg/kg/d: 100% success rate; 15 mg/kg/d: 80% success rate). We chose to administer a consistent dose of opioids to the dam throughout pregnancy and lactation to model the current management of pregnant mothers with opioid use disorder. Women with opioid use disorder are now encouraged to enroll in opioid medication-assisted treatment programs with the goal of attaining a steady-state drug level. Maintaining a consistent level of opioid administration during pregnancy in mothers with opioid use disorder limits adverse maternal and fetal consequences associated with fluctuations related to higher opioid concentrations. Increases in opioid dosage can result in maternal respiratory depression and lethal overdose and may decrease fetal heart rate and variability (Krans et al., 2015; Rosenthal and Baxter, 2019).

#### Surgery and Drug Delivery System

Female dams were anesthetized at P95 with isoflurane (5% induction, 2% maintenance, 0.5 l/min) and placed in the mouse adapter (Stoelting, Wood Dale, IL, United States). Body temperature was maintained at 37°C using a heating pad. The dorsum of the back was shaved and a ∼1 cm horizontal incision was made below the scapulae with subsequent formation of a subcutaneous pocket. The Alzet pump was implanted and continuously infused with either Oxy or sterile 0.9% NaCl (Veh) over a period of 60 days. The pump duration allowed for adequate post-surgical recovery time, breeding, and administration of treatment through weaning of offspring at P21. In addition, the use of a subcutaneous pump limited unwanted maternal stress that can occur with daily injections.

### Behavioral Testing

#### Maternal Isolation-Induced Ultrasonic Vocalization Recording

Neonates with NAS often exhibit excessively high-pitched crying, irritability, and prolonged periods of inconsolability (Anbalagan and Mendez, 2020). Affective characteristics of ultrasonic vocalizations (USVs) in rodents are generally thought to communicate different emotional states, such as aggression or pain. USV quantity, duration, pitch, frequency, and loudness (dB) of the calls allow for the assessment of call characteristics following *in utero* Oxy exposure (Vivian and Miczek, 1993a,b). USV recordings were performed on P5, P7, P9, and P11 (Figure 1A). Dams were removed from the home cage and placed into a clean IVC for the duration of testing. The home cage with the pups in nest was placed into a warming box (Harvard Apparatus) set to 34°C for 10 min prior to the start of testing. We maintained an average pup surface body temperature of 34°C prior to placement into the USV recording chamber, as low pup body temperature increases USV production (Branchi et al., 2001). The surface body temperature of all pups was assessed via a non-contact HDE Infrared Thermometer prior to placement into the recording chamber, and no differences in body surface temperature were observed between groups. The recording chamber was maintained at room temperature (22–23°C). For recording, pups were individually removed from the home cage and placed into an empty standard mouse cage (28.5 × 17.5 × 12 cm) inside a sound-attenuating chamber (Med Associates). USVs were recorded via an Avisoft UltraSoundGate CM16 microphone placed 5 cm away from the top of the cage, Avisoft UltraSoundGate 116H amplifier, and Avisoft Recorder software (gain = 3 dB, 16 bits, sampling rate = 250 kHz). Pups were recorded for 3 min, after which they were weighed and returned to home cages. Frequency sonograms were prepared from USV recordings in MATLAB [frequency range = 25–120 kHz, Fast Fourier Transform (FFT) size = 512, overlap = 50%, time resolution 1.024 s, frequency resolution = 488.2 Hz]. Individual calls and other spectrotemporal features were identified from the sonograms adapted from validated procedures (Holy and Guo, 2005; Maloney et al., 2018a,b).

#### Developmental Reflexes and Milestones Assessment

Mice were evaluated for achievement of physical and behavioral milestones from early development through early juvenile stage. Weight was measured at 10 time points: P5, P7, P9, P11, P14, P23, P25, P27, and P30. A visual inspection of normal physical milestone attainment was performed with evaluation for detached pinnae at P5 and eye opening at P14. Righting reflex was assessed at P14 as follows: each mouse was placed prone onto its abdomen and quickly pronated 180° to its back in a smooth motion. The time for the mouse to right itself with all four paws positioned underneath the abdomen was recorded (Zanni et al., 2020). Each mouse underwent three timed trials, which were averaged for analysis.

### Statistical Analyses

SPSS (IBM, v.25) was used for all statistical analyses. Data were screened for missing values, influential outliers, fit between distributions and the assumptions of normality and homogeneity of variance. Variables that violated assumptions of normality (including number of USV calls, mean pitch, pitch range, and peak power) were square root-transformed. Data were analyzed using hierarchical linear models with sex clustered within litters and age clustered within individual pups. Fixed factors were dam, drug, sex and, where appropriate, age. Age was also treated as a random repeated effect and was grand mean-centered for analysis. Interactions between the fixed factors are reported when significant. If an interaction effect was significant, *p*-values were obtained from the hierarchical linear model for simple main effects and reported for differences between different levels within the interaction. If sex had a significant main effect, findings are shown segregated by sex. As litter size can influence behavior and litter cannot be separated from drug treatment in this study, all models included litter size as a covariate. Probability value for all analyses was *p* < 0.05. Test statistics and analysis details are provided in Table 2. The datasets generated for this study are available upon reasonable request to the corresponding author.

**TABLE 2:** Test statistics from hierarchical linear models.

Variable	Factor	Output	*p*-value
Weight (g)	Sex	*F* (1, 107) = 10.219	*p* = 0.002
	Drug	*F* (1, 109) = 5.448	*p* = 0.021
	Age	*F* (9, 901) = 785.001	*p* = 0.000
	Litter Size	*F* (1,102) = 9.922	*p* = 0.002
	Sex × Dam × Drug × Age	*F* (67, 685) = 4.232	*p* = 1.2916E^–22^
Righting reflex	Dam	*F* (1,24) = 4.630	*p* = 0.042
	Sex × Dam × Drug	*F* (4,74) = 2.518	*p* = 0.048
	Litter Size	*F* (1,26) = 9.335	*p* = 0.005
Number of USV calls	Dam	*F* (1,156) = 7.015	*p* = 0.009
	Drug	*F* (1,151) = 12.000	*p* = 0.001
	Dam × Drug	*F* (1,159) = 5.223	*p* = 0.024
	Litter Size	*F* (1, 159) = 3.064	*p* = 0.082
Pitch range (Hz)	Drug	*F* (1,169) = 8.456	*p* = 0.004
	Dam × Drug	*F* (1,169) = 13.528	*p* = 0.000315
	Age × Dam × Drug × Sex	*F* (24, 319) = 1.385	*p* = 0.097
Mean pitch (Hz)	Drug	*F* (1,160) = 29.552	*p* = 1.9953E^–7^
	Dam × Sex	*F* (1,160) = 5.373	*p* = 0.022
	Drug × Sex	*F* (1,160) = 5.354	*p* = 0.022
	Sex × Dam × Drug	*F* (1,160) = 8.365	*p* = 0.004
	Sex × Dam × Drug × Age	*F* (24, 301) = 1.617	*p* = 0.036
	Sex	*F* (1, 161) = 3.190	*p* = 0.076
Peak power (dB)	Dam	*F* (1,163) = 5.632	*p* = 0.019
	Drug	*F* (1,171) = 10.327	*p* = 0.002
	Dam × Drug	*F* (1,172) = 12.399	*p* = 0.001
	Litter Size	*F* (1,161) = 6.313	*p* = 0.013

*Showing significant main and interaction effects. Age was grand mean-centered for analysis, where included. Litter size was included as a covariate.*

## Results

### Oxycodone Impacted Developmental Weight Trajectories Differentially by Sex and Exposure Duration

**FIGURE 2:**
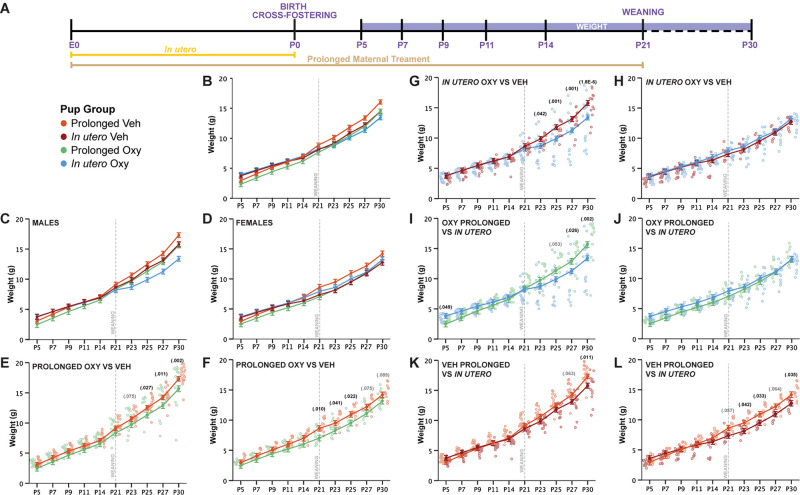
Prolonged and *in utero* Oxy exposure, as well as cross-fostering, decreases weight gain after weaning. **(A)** Schematic of the treatment paradigm for maternal Oxy exposure and weight measurements throughout development. **(B)** Line graph of weight in all offspring (sex, *p* = 0.002; drug, *p* = 0.021; age, *p* < 0.000; sex × dam × drug × age, *p* = 1.2916E^– 22^). **(C,D)** Line graph of weight in **(C)** male and **(D)** female offspring. **(E,F)** Prolonged Oxy exposure, relative to prolonged Veh exposure, led to significantly lower weights post-weaning in **(E)** male (P23, *p* = 0.075; P25, *p* = 0.027; P27, *p* = 0.011; P30, *p* = 0.002) and **(F)** female offspring (P21, *p* = 0.010; P23, *p* = 0.041; P25, *p* = 0.022; P27, *p* = 0.075; P30, *p* = 0.089). **(G,H)** *In utero* Oxy exposure, relative to *in utero* Veh exposure, led to significantly lower weights post-weaning in **(G)** male offspring only (P23, *p* = 0.042; P25, *p* = 0.001; P27, *p* = 0.001; P30, *p* = 0.000018), with no effects in **(H)** female offspring. **(I,J)** *In utero* Oxy exposure with cross-fostering, relative to prolonged Oxy exposure, led to decreased weight in adolescence in **(I)** male pups only (P5, *p* = 0.049; P25, *p* = 0.053; P27, *p* = 0.026; P30, *p* = 0.002), with no effects in **(J)** female offspring. **(K,L)** *In utero* Veh exposure with cross-fostering, relative to prolonged Veh exposure, led to decreased weight post-weaning in **(K)** male (P27, *p* = 0.063; P30, *p* = 0.011) and **(L)** female offspring (P21, *p* = 0.057; P23, *p* = 0.042; P25, *p* = 0.033; P27, *p* = 0.064; P30, *p* = 0.035). Closed circles depict mean weight, with litter size as a covariate (*p* = 0.002), while open circles depict individual weights. Gray vertical line indicates date of weaning.

We examined the effects of Oxy administration on gross and sensorimotor development in mice from birth throughout the early juvenile stage (Figures 2A,B). To evaluate general health and gross development, we assessed the appearance of physical milestones and weight. No differences were observed between groups for pinnae detachment at P5 or eye opening by P14. In our analysis of weight, we found male mice weighed significantly more than females in all groups at all ages, and therefore, weight data are segregated by sex (Figures 2C,D) from the full factorial linear mixed model including sex, drug, and duration as factors.

Prolonged Oxy exposure led to an overall decrease in mean weight compared to prolonged Veh exposure, which was more pronounced in male offspring. Prolonged Oxy-exposed male offspring exhibited significantly reduced weights compared to prolonged Veh offspring post-weaning at P25, P27, and P30, with non-significant reductions at P23 (Figure 2E). Female prolonged Oxy offspring showed significantly reduced weight compared to prolonged Veh controls at P21, P23, and P25, with non-significant reductions at P27 and P30 (Figure 2F). These data indicate that overall prolonged Oxy exposure reduces weight across development in male and female offspring, with the effect on weight gain compounding once potentially compensatory maternal care is lost after weaning.

We then evaluated the potential effects of early opioid cessation (*in utero* Oxy) on weight gain in male and female offspring and once more found male offspring susceptible to Oxy effects. In contrast to prolonged exposure, an overall reduction in weight was not observed with *in utero* Oxy exposure compared to *in utero* Veh exposure. However, *in utero* Oxy males showed a precipitous decrease in weight gain trajectory after weaning from the foster dam at P23, P25, P27, and P30 as compared to *in utero* Veh controls (Figure 2G). Female *in utero* Oxy offspring showed no difference in weight across development compared to *in utero* Veh controls (Figure 2H). Clinically, male infants are more susceptible to NAS (Charles et al., 2017), so the precipitous decrease in weight gain trajectory in the *in utero* Oxy male offspring may be associated with withdrawal symptomatology unmasked by cessation of care under a foster dam.

We also examined weight trajectories between *in utero* and prolonged vehicle-exposed groups. In vehicle-exposed males, cross-fostering was associated with decreased weights in the *in utero* Veh group at P30, with a trend toward decreased weight at P27, relative to the prolonged Veh group (Figure 2K). Of interest, cross-fostered female pups (*in utero* Veh) weighed less compared to prolonged Veh female pups post-weaning at P23, P25, and P30 (Figure 2L). These findings suggest that cross-fostering alone can influence post-weaning weight trajectories in a sex-dependent manner. However, it is noteworthy that the decrease of weight gain trajectories in the male *in utero* Oxy group persisted above and beyond the observed decreased weights in male *in utero* Veh controls, indicating *in utero* Oxy exposure affects weight gain when controlling for cross-fostering (Figure 2G).

We have shown so far that Oxy exposure, compared to Veh, decreased post-weaning weight following both long and short exposures. We next sought to determine how the duration of Oxy exposure influences weight by assessing the weight gain trajectory differences between prolonged and *in utero* Oxy pups. Comparisons between the male mice showed *in utero* Oxy pups initially weighed more than prolonged Oxy pups at P5. However, after the *in utero* Oxy male pups were weaned at P21, their weights decreased relative to the prolonged Oxy group at P27 and P30 (Figure 2I). No differences in weight gain between *in utero* and prolonged groups were detected in female Oxy-exposed pups (Figure 2J). Overall, early Oxy cessation was associated with increased weights at very early post-natal ages, followed by weight loss in males at weaning.

### Prolonged Oxycodone Exposure Altered Sensorimotor Reflex in Female Offspring Only

**FIGURE 3:**
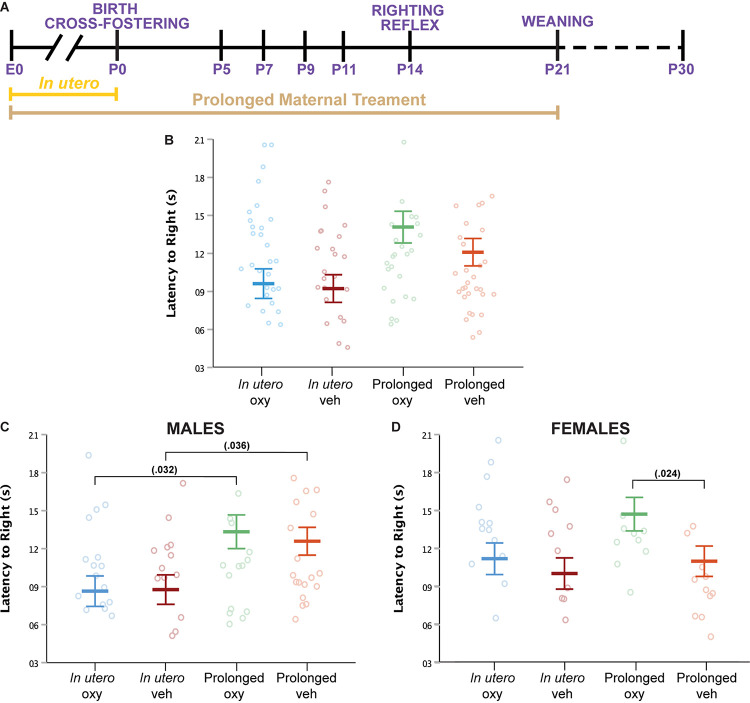
Oxy exposure delays maturing of sensorimotor reflexes. **(A)** Schematic of the treatment paradigm for maternal Oxy exposure and righting reflex measurement throughout development. **(B)** Mean latency to right in all offspring (sex × dam × drug × age, *p* = 0.048; dam, *p* = 0.042). **(C,D)** Mean latency to right in **(C)** male and **(D)** female offspring. Prolonged Oxy exposure led to significantly longer latency to right relative to *in utero* Oxy (*p* = 0.032) in **(C)** male pups and relative to prolonged Veh (*p* = 0.024) in **(D)** female pups. Male pups exposed to prolonged Veh also show longer latency to right relative to *in utero* Veh (*p* = 0.036). Mean latencies, with litter size as a covariate (*p* = 0.005), while open circles depict individual latencies. Error bars represent standard error.

Righting reflex at P14 was examined as an assessment of sensorimotor milestones, early gross locomotor abilities, and general strength (Figures 3A,B). In males, no difference in latency to right was noted between the prolonged Oxy or prolonged Veh groups (Figure 3C). An increased latency to right was demonstrated in prolonged Oxy male pups compared to *in utero* Oxy. However, cross-fostering may have a confounding effect on the righting reflex in male pups, since prolonged Veh males also exhibited an increased latency to right relative to *in utero* Veh males. In females, prolonged Oxy pups exhibited significantly increased latency to right relative to prolonged Veh controls (Figure 3D). No significant differences in the righting reflex were observed in the *in utero* Oxy or *in utero* Veh female offspring. Together, these data indicate that females, but not males, are susceptible to the effects of prolonged developmental Oxy exposure on attainment of the sensorimotor reflex.

### Oxycodone Exposure Disrupts Early Communicative Behaviors

**FIGURE 4:**
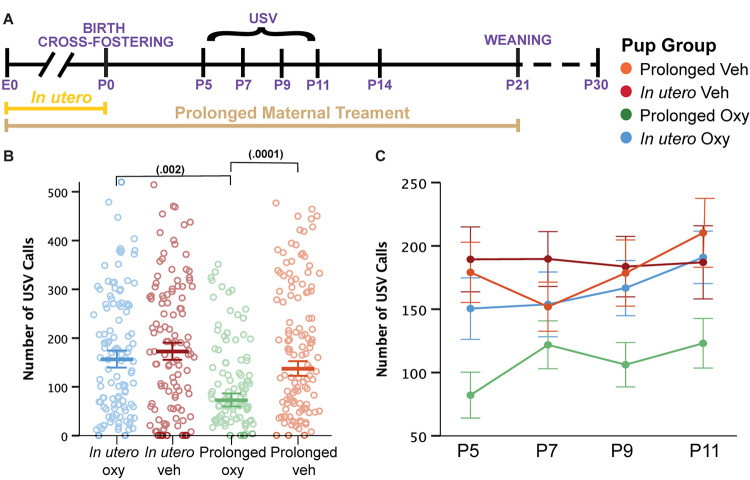
Prolonged Oxy exposure decreases pup USV call production. **(A)** Schematic of the treatment paradigm for maternal Oxy exposure and USV measurements throughout development. **(B)** Cumulative means number of USV calls (dam, *p* = 0.009; drug, *p* = 0.001; dam × drug, *p* = 0.024). Prolonged Oxy exposure led to decreased number of calls relative to prolonged Veh (*p* = 0.000126) and relative to *in utero* Oxy exposure (*p* = 0.002). **(C)** Line graph of mean call number at all time points. Thick bars and closed circles depict mean call number, with litter size as a covariate, and open circles depict individual call numbers. Error bars represent standard error.

Language delays have been demonstrated in toddlers with prenatal opioid exposure (Conradt et al., 2019). Therefore, we assessed early affective and communicative behaviors by evaluating maternal isolation-induced USVs. USVs are an affective and communicative response that elicits maternal search and retrieval, lactation, and caretaking behaviors (Haack et al., 2009; Maloney et al., 2018a). As a result, characterization of quantity and quality of USV calls has been used in the rodent literature as a model for investigating early communicative deficits (Enga et al., 2016). Here, we quantified USV production and spectrotemporal features to examine the influence of Oxy on early communicative behaviors during the first 2 weeks of life (Figure 4A). Overall, we detected a highly significant effect of continued Oxy exposure on USV production. Specifically, prolonged Oxy pups produced significantly fewer USVs relative to prolonged Veh pups and *in utero* Oxy pups (Figure 4B), which persisted from P5 through P11 (Figure 4C), an age at which the C57Bl/6J strain used here still has a detectable call rate (Rieger and Dougherty, 2016). While we did not see as substantial of a peak at P7/P9 as we normally see in these experiments, the effect of prolonged Oxy was consistent regardless.

**FIGURE 5:**
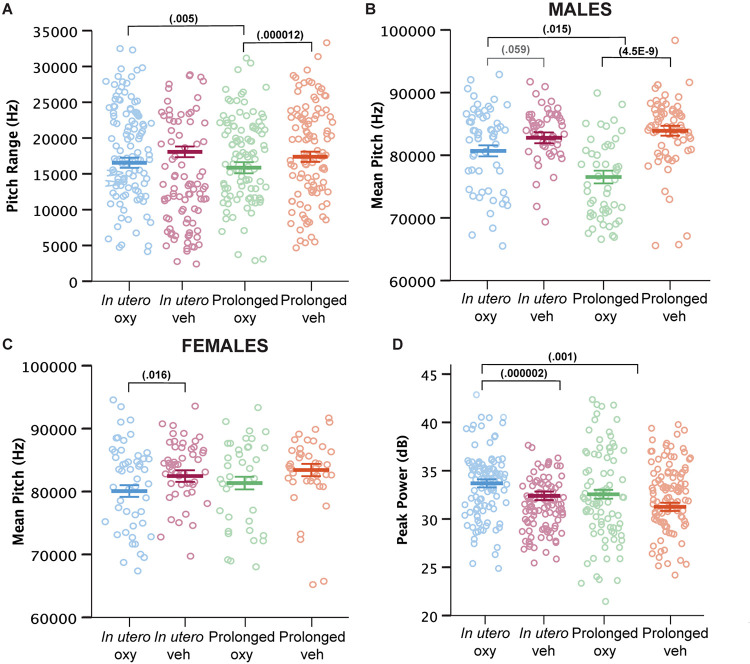
Prolonged and *in utero* Oxy exposure affect spectrotemporal features of pup USV calls. **(A)** Cumulative means of pitch range (Hz) of USV calls (drug, *p* = 0.004; dam × drug, *p* = 0.000315; sex × dam × drug × age, *p* = 0.097). Prolonged Oxy exposure led to lower pitch range relative to prolonged Veh (*p* = 0.000012) and relative to *in utero* Oxy exposure (*p* = 0.005). **(B,C)** Cumulative mean pitch (Hz) in **(B)** male and **(C)** female offspring (drug, *p* = 1.9953E^– 7^; sex × dam, *p* = 0.022; sex × drug, *p* = 0.022; sex × dam × drug, *p* = 0.004; sex × dam × drug × age, *p* = 0.036; sex, *p* = 0.076). Prolonged Oxy exposure in **(B)** male pups led to decreased pitch relative to prolonged Veh (*p* = 4.4816E^– 9^) and relative to *in utero* Oxy (*p* = 0.015). *In utero* Oxy exposure led to a marginal decrease in pitch relative to *in utero* Veh (*p* = 0.059) in **(B)** male pups and a significant decrease in **(C)** female pups (*p* = 0.016). **(D)** Cumulative means of peak power (dB) (dam, *p* = 0.019; drug, *p* = 0.002; dam × drug, *p* = 0.001). *In utero* Oxy exposure leads to significantly higher peak power relative to prolonged Oxy exposure (*p* = 0.001) and relative to *in utero* Veh (*p* = 0.000002). Thick bars depict means, with litter size as a covariate, while open circles depict individual measurements. Error bars represent standard error.

Beyond call numbers, spectrotemporal USV features such as duration, pitch frequency, and power (loudness) inform of an affective component to USV characteristics (Wöhr and Schwarting, 2013). In previous analyses of USV spectrotemporal features in mouse models of intellectual and developmental disorder risk factors and early drug exposure models, we and others have demonstrated the vulnerability of these features to genetic and early environmental insults (Dougherty et al., 2013; Maloney et al., 2018a,b; Kopp et al., 2019). We examined call features including call duration, pitch range and mean, peak power, and fraction of calls with a pitch jump. Prolonged Oxy administration narrowed the USV pitch range compared to USVs produced by *in utero* Oxy pups and prolonged Veh controls (Figure 5A). Prolonged Oxy exposure also led to a highly significant reduction in mean pitch of USVs in prolonged Oxy male pups compared to *in utero* Oxy and prolonged Veh males (Figure 5B). Interestingly, USVs produced by prolonged Oxy female pups did not show a significant difference in pitch compared to prolonged Veh females (Figure 5C). However, female *in utero* Oxy offspring did exhibit USVs with significantly lower mean pitch relative to *in utero* Veh. A similar non-significant reduction in mean pitch was observed in USVs produced by *in utero* Oxy males relative to *in utero* Veh males (Figure 5B). Thus, *in utero* Oxy exposure was associated with changes in affective components of communication, the significance of which warrants further investigation. Since opioid withdrawal has been associated with high-pitched crying and increased agitation, we also assessed for alterations in USV peak power. *In utero* Oxy exposure resulted in louder USV calls compared to those produced by prolonged Oxy pups and *in utero* Veh controls (Figure 5D). The increased peak power, or loudness, in *in utero* Oxy pup calls only may be temporally related to onset of withdrawal after drug cessation at P0, relative to the prolonged Oxy pups which are weaned off the drug at P21.

Overall, prolonged Oxy pups demonstrated significant decreases in number of USVs along with a narrower pitch frequency range and mean pitch in male pups. *In utero* Oxy pups produced a similar number of USVs compared to controls, yet those calls were louder than controls and prolonged Oxy calls, and lower in mean pitch when produced by females. Together, our ontogenetic model of *in utero* Oxy exposure demonstrates some alterations in loudness of affective calls, while the prolonged Oxy exposure further shows alterations in number and spectrotemporal features of early communicative and affective behaviors.

## Discussion

Here we present a novel model to investigate the ontogenetic impact of *in utero* only versus prolonged mitigating opioid exposure on early neurodevelopmental outcomes, while controlling for confounding factors present in clinical observational studies. The utilization of a biological dam and cross-foster dam in our novel model of ontogenetic rodent exposure was based on an attempt to parallel opioid exposure through a method most consistent with clinical management and observations. Active maternal bonding and breast-feeding has been shown to decrease hospitalization length, decrease rates of pharmacotherapy administration, and decrease NAS severity in the NICU (Rosenthal and Baxter, 2019). The prolonged Oxy group remained with the biological dam and was weaned off Oxy through lactation, allowing for the assessment of continued post-natal exposure on early development. However, many infants with NAS experience decreased bonding time and skin–skin contact with the biological mother secondary to socioeconomic barriers and are cared for by healthcare staff for NAS. This type of setting may result in an environmental stressor to the neonate due to inconsistent maternal contact, frequent alteration of caregivers, along with a higher incidence of foster care placement following hospital discharge in neonates with maternal history of drug use (Brundage and Levine, 2019). As a result, we rationalized a cross-foster approach would be a feasible model for evaluating the effects of *in utero* opioid exposure on developmental impact in the *in utero* Oxy group.

*In utero* Oxy exposure decreased weight gain trajectory following weaning from foster dams, in male offspring. Further, *in utero* Oxy-exposed male and female pups showed alterations in the spectrotemporal features of USVs. Meanwhile, offspring with prolonged Oxy exposure until weaning at P21 demonstrated poorer neurodevelopmental outcomes compared to mice exposed only until birth. Notably, continued post-natal Oxy exposure was associated with decreased weight gain trajectory, impaired motor reflexes, and abnormal early communication behaviors. Both male and female offspring in prolonged Oxy exposure groups had decreased weight gain following weaning at P21, with delayed latency to right observed in females. In addition, prolonged Oxy-exposed offspring had significantly reduced USV production and alterations in spectrotemporal features reflecting affective and early communicative impairment.

### Oxycodone Exposure Impairs Attainment of Physical and Motor Development

Decreased fetal growth can be used as a general indicator of harmful *in utero* drug exposures (Haight, 2018). The association between birth weight and decreased infant survival is highly robust, though the underlying biological mechanisms are not always clearly understood (Basso et al., 2006; Yazdy et al., 2015). We did not obtain birth weights at P0 in order to minimize animal handling which can reduce behavioral and hormonal reactivity to stress and confound behavioral testing results (Luchetti et al., 2015). Initial weight assessment occurred at P5 with no observed effect of *in utero* or prolonged Oxy exposure relative to Veh controls. Overall, human literature shows low birth weight in the setting of maternal methadone use during pregnancy, but no evidence of low birth weight following *in utero* exposure to other opioids including codeine, tramadol, hydrocodone, or Oxy (Yazdy et al., 2015). Thus, our findings are consistent with existing Oxy human literature that shows no reported association between Oxy and low birth weight in neonates (Kelly et al., 2011; Yazdy et al., 2015).

Interestingly, female offspring exposed to continued Oxy, relative to Veh controls, showed a significant decrease in weight after weaning at P21, which persisted through P25, early juvenile development in mice. Neurodevelopmental processes occurring at these ages in the rodent occur in the human at approximately 2–3 years and pre-pubertal juvenile ages, respectively (Semple et al., 2013). Male weights, after prolonged exposure, showed a significant decline on P25–P30 as compared to Veh controls. Since these offspring were separated from the dam partially through a period during which they are naturally weaning from nursing (König and Markl, 1987), it is unclear whether the acute onset of decreased weight gain after separation from the dam at P21 is related to Oxy withdrawal symptomatology or not. It is certainly plausible. However, human studies of prenatal opioid exposure have described decreased adaptive behaviors during infancy through toddlerhood (Conradt et al., 2019). Cessation of care from the biological dam may potentially have uncovered deficiencies in self-care of offspring. The decreased weight gain post-weaning could also stem from maladaptive feeding behaviors secondary to Oxy exposure, since the opioid system has a strong role driving food intake homeostasis (Valbrun and Zvonarev, 2020).

Early Oxy cessation significantly decreased weight gain trajectory in a sex-specific manner not observed in the prolonged Oxy exposure cohort. Interestingly, *in utero* Oxy exposure led to significantly higher weight at P5 in males, though both groups show comparable averages at P21. Female weights following *in utero* Oxy exposure do not show any significant weight differences from controls. Males exposed to Oxy *in utero* showed a rapid, significant and persistent decrease in weight gain following weaning. Since the *in utero* exposure group was cross-fostered at birth to a non-drug exposed dam, normal weight gain trajectory was potentially maintained through adequate maternal care from the foster dam. A further explanation could be the “two-hit” hypothesis in which early life susceptibility, such as abstinence and withdrawal following *in utero* Oxy exposure, compounded with the post-natal stress of weaning precipitated a weight loss phenotype (Nederhof and Schmidt, 2012; Peña et al., 2019). Perhaps, males are more sensitive to early life stressors and may have long-term consequences from *in utero* opioid exposure compared to females. Male human neonates are more at risk for developing NAS compared to females, so long-term changes in opioid circuitry governing feeding behaviors could explain the abnormal weight trajectory in male mouse offspring post-weaning (Charles et al., 2017). Prospective human studies evaluating the long-term effects of *in utero* opioid and effects on weight trajectory during childhood through adulthood have not been performed to our knowledge. The altered weight trajectory findings in both the *in utero* Oxy and prolonged Oxy suggest a potential role of *in utero* opioid exposure on long-term impact on growth that requires further evaluation in the human literature.

In the vehicle-treated groups, cross-fostered pups showed decreased weight gain trajectory after weaning relative to pups reared by a biological dam. Our observation of decreased weight gain following weaning in Veh-exposed cross-fostered pups may be related to potential alterations in emotionality and stress responses secondary to confounders involved with cross-fostering, such as early handling (Luchetti et al., 2015). Regardless, the effect of cross-fostering on weight did not mask our ability to identify effects of *in utero* Oxy exposure on weight in males. Indeed, the effect of *in utero* Oxy exposure on weight occurred at additional younger ages and with a larger magnitude than *in utero* Veh exposure and persisted when controlling for effects of litter and cross-fostered dam status. Similarly, in females, weight reduction was observed following prolonged Oxy exposure compared to prolonged Veh controls, and *in utero* Veh exposure compared to the prolonged Veh exposure control group. Despite the independent effect on weight by cross-fostering, this method was valuable in allowing us to cease Oxy exposure at birth and thus observe effect of Oxy limited to *in utero* development. Furthermore, these findings highlight the importance of including proper cross-foster control groups in study designs for accurate interpretation of results.

Prenatal opioid exposure has also been associated with delays in attainment of motor milestones in children. A meta-analysis by Yeoh et al. (2019) detected significant delays in motor outcomes in children aged 0–6 years that experienced prenatal opioid exposure. We assessed the righting reflex at P14, the beginning of the visual critical period, and an age at which mice should be fully ambulatory (Williams and Scott, 1954; Hooks and Chen, 2007; Feather-Schussler and Ferguson, 2016). The righting reflex corrects the orientation of the body from an off axis position (Troiani et al., 2005). Proper execution of the reflex requires a combination of visual, vestibular, and somatosensory system inputs to make appropriate postural adjustments through neural pathways within the brain and cerebellum. Females in the prolonged Oxy exposure group had significantly increased latency to right compared to Veh controls. There was no significant difference in righting reflex latency between females exposed to Oxy and Veh *in utero*. Hence, only continued post-natal Oxy exposure seems to result in delayed sensorimotor development. Previously, increased latency to right has been demonstrated with *in utero* morphine exposure in both male and female rat pups, but rodent studies evaluating effects of opioids on the righting reflex have been limited (Slamberová et al., 2005). Though the exact mechanism of action is unclear, significant evidence in the literature demonstrates selective vulnerability of cerebellar granule neuroblasts to opioids, along with opioids’ negative effects on neuronal somatosensory cortex development (Seatriz and Hammer, 1993; Hauser et al., 2003). Multiple studies have further linked opioid exposure to increased apoptosis and decreased differentiation of Purkinje cells in the cerebellum (Hauser et al., 2003). Additionally, perinatal morphine treatment in rats decreased total number of neurons in the somatosensory cortex (Seatriz and Hammer, 1993). Thus, it is possible multiple circuits are mediating the effect. Overall, the sex-specificity of the effect is also interesting. This observed sex bias could be related to sex-specific dimorphic alterations in catecholamine levels in the cerebellum as shown in a previous study of prenatal morphine exposure (Vathy et al., 1995). Interestingly, cross-fostering resulted in a shorter latency to exhibit the righting reflex in males compared to male pups reared by biological dams. This reflex is dependent on vestibular inputs that sense head movement but lacks cortical involvement. The righting reflex is frequently used in studies evaluating anesthesia reversal, sepsis survival or traumatic brain injuries (Gao and Calderon, 2020). In general, an increased latency to right is associated with decreased arousal or underlying neurological impairment but a decreased latency has not been shown to correlate with any particular pathophysiological condition or stressed anxiety state (Gao and Calderon, 2020). Decreased latency to right is unlikely to be linked to a behavioral or neurological impairment but could be secondary to a non-pathological increased arousal state due to cross-fostering in these litters. Overall, the shorter latency seen to right in the cross-fostered group is likely not indicative of altered development of sensorimotor reflexes. However, future studies will be necessary to delineate the mechanisms underlying this behavioral phenotype, and its sex-specific expression.

### Ontogenetic Oxycodone Exposure May Delay Early Communicative Behaviors and Alter Spectrotemporal Features of USVs

Currently, studies exploring the effects of prenatal opioid exposure on language development in children demonstrate equivocal results (Conradt et al., 2019). Previous work has identified language development impairments following *in utero* exposure to methadone or heroin (Conradt et al., 2019). However, several of these analyses did not control for important confounders such as socioeconomic status or maternal use of other substances. In general, large epidemiological studies evaluating impact of prenatal opioid exposure on language development have been difficult to perform due to various confounding environmental variables including quality of caregiving, parental education level, and socioeconomic factors. Despite the advantage of limiting confounds through the use of rodent models, and indications that communicative circuits are conserved between rodents and humans (Arriaga et al., 2012), there have been minimal rodent studies evaluating the effects of prenatal opioid exposure on early communicative behaviors to date. Isolation-induced USVs are a strongly conserved adaptive response of young rodent pups to elicit maternal caregiving responses (Haack et al., 2009). Our observed collective decrease in mean USV production following prolonged Oxy exposure, compared to *in utero* Oxy exposure and Veh controls, is suggestive of impaired early communication. Previous studies have shown evidence for neuropharmacological modulation of USVs through alteration of mood or arousal state (Vivian and Miczek, 1991, 1993b; Maloney et al., 2018a). In addition to serving as an analgesic, Oxy is also a sedative and an anxiolytic agent, which may decrease USV calling in the prolonged Oxy exposure pups by actively suppressing USV circuitry secondary to reduced reactivity to surrounding environmental stressors and decreased respiratory rate (Rao and Desai, 2002). Furthermore, the interplay between pup communication and maternal care is complicated. In vocally impaired pups, decreased USV production has been shown to result in maternal neglect, because without calls the dams cannot locate the pups outside of the nest (Hernandez-Miranda et al., 2017). Thus, maternal care may be reduced in response to decreased USVs calling by prolonged Oxy-exposed pups. Maternal care has also been reported to be attenuated following morphine exposure during pregnancy, with a study reporting increased time to pup retrieval, decreased nursing and cleaning of pups, and increased maternal self-care time (Slamberová et al., 2001). This reduced maternal care could further disrupt neurodevelopment of the pup, and thus be a possible indirect influence on later adult behaviors. However, currently the literature on maternal care following Oxy exposure is conflicting. Two recent rodent studies found no changes in maternal behavior or maternal motivation following dam Oxy exposure (Watters et al., 2020; Zanni et al., 2020). Notably, in our study, *in utero* Oxy exposed offspring demonstrated comparable USV means to Veh controls. Normal USV call production in the *in utero* Oxy offspring at P5 suggests that, in the prolonged exposure group, Oxy reduces USV production by acute suppression of USV circuits. Finally, the adequate weight trajectories before weaning in the Oxy group further indicate appropriate dam care. Hence, we would hypothesize that maternal care likely did not result in the behavioral deficits observed. Still, to our knowledge, the direct impact of Oxy exposure on maternal behaviors has not been examined. Furthermore, there is currently scant literature considering the effect of ongoing Oxy exposure on maternal behavior in rodents, with even more limited studies utilizing a mouse model. This warrants an individual study to assess reciprocity of interactions between pup and dam. Overall, deficits in observed USV production could be the result of a combination of factors, including acute drug effects on circuits, influence of dam care, and opioid-mediated effects on neural development and communication.

Affective characteristics of USVs in rodents are generally thought to communicate different emotional states, such as aggression or pain. Rodent USVs are particularly interesting as they occur only in salient situations such as exposure to painful stimuli, maternal behavior, sexual behavior, or aggression. As has been well-characterized in rats, affective features of rodent USVs may be reflected by alterations in duration, pitch, frequency, and loudness (dB) of the calls (Vivian and Miczek, 1993a,b). In our developmental cohort, pups exposed to prolonged Oxy demonstrated decreased mean pitch (in males only), and narrower pitch range. Interestingly, a previous study administered morphine to adult rats and observed decreased USV pitch, duration, and rate (Vivian and Miczek, 1993a). The decreased pitch and pitch range could be a result of Oxy’s depressive effects on respiration, or of Oxy’s anxiolytic drug properties which may dampen USV circuity. For the *in utero* Oxy exposure group, we predicted increased USV production along with increased duration, pitch, frequency, and amplitude of USVs secondary to discomfort associated with withdrawal. The assumption is based on the current human description of NAS characterized by human neonates exhibiting prolonged periods of high-pitched crying and inconsolability secondary to withdrawal (Anbalagan and Mendez, 2020). However, human studies do not formally characterize the spectrotemporal features of crying in infants with NAS, so the description of a high-pitched cry may be subjective in nature. We found that that continued opioid exposure during the post-natal period in the prolonged Oxy group resulted in less USV calls compared to both the *in utero* Oxy and vehicle controls. We did not expect the prolonged Oxy group to be undergoing withdrawal at this time, thus we did not hypothesize an increase in call rate in this group. A reduction in call rate is likely more consistent with an acute dampening of the circuits mediating USV production by the continued presence of Oxy in these pups during recordings. Indeed, reduced call rates were observed in rat pups following injection of μ- or δ-opioid receptor agonists (Carden et al., 1991). Future studies are needed to evaluate the impact of early suppression of these social communicative circuits by opioid agonists on social behavior consequences at older ages.

Our novel Oxy administration paradigm enables future exploration of withdrawal periods, to determine if NAS following Oxy cessation can be appropriately modeled in rodents. If so, testing of novel agents for treatment of withdrawal symptoms will be possible, with the goal of limiting continued post-natal opioid exposure given potential long-term side effects of early post-natal opioid administration on neurodevelopment (Attarian et al., 2014). Finally, few mouse models of *in utero* opioid exposure currently exist, with the majority of the literature utilizing rat perinatal opioid models. Thus, our model will facilitate genetic manipulations using established cutting-edge genetic tools available in the mouse to broaden understanding of the mechanisms mediating consequences of early opioid exposure on neurodevelopment.

## Data Availability Statement

The raw data supporting the conclusions of this article will be made available by the authors, without undue reservation.

## Ethics Statement

The animal study was reviewed and approved by the Institutional Animal Care and Use Committee of Washington University in St. Louis.

## Author Contributions

EM contributed to the conceptualization, methodology, investigation, data curation, writing (original draft preparation and editing), and funding acquisition for this project. SS contributed to the methodology, validation, formal analysis, writing (original draft preparation and editing), and visualization for this project. MM, KB, and KM contributed to the methodology for this project. JD contributed to the conceptualization, methodology, validation, resources, writing (draft editing), supervision, project administration, and funding acquisition for this project. RA-H contributed to the conceptualization, methodology, validation, writing (draft editing), supervision, project administration, and funding acquisition for this project. SM contributed to the conceptualization, methodology, data curation, resources, writing (draft editing), validation, supervision, project administration, and funding acquisition for this project. All authors contributed to the article and approved the submitted version.

## Conflict of Interest

The authors declare that the research was conducted in the absence of any commercial or financial relationships that could be construed as a potential conflict of interest.
